# Numerical Analysis of Broadband Noise Generated by an Airfoil with Spanwise-Varying Leading Edges

**DOI:** 10.3390/biomimetics9040229

**Published:** 2024-04-11

**Authors:** Lei Wang, Xiaomin Liu, Chenye Tian, Dian Li

**Affiliations:** 1 Department of Fluid Machinery and Engineering, School of Energy and Power Engineering, Xi’an Jiaotong University, Xi’an 710049, China; 2 School of Energy and Power Engineering, Xi’an University of Technology, Xi’an 710048, China

**Keywords:** aerodynamic noise, serration structure, the leading edge of the airfoil, noise suppression, numerical prediction

## Abstract

Here, the single-target parameterization of alternatives to leading-edge noise is carried out using analytical models based on the Wiener–Hopf technique. Four leading-edge serration profiles with different frequencies, amplitudes, and phases are implemented to aid the understanding of sound suppression mechanisms. The effects of the serrated shape factor, wavelength, and amplitude are analyzed at tip-to-root ratios of 0.5, 1, and 2, respectively. An effective double-wavelength sinusoidal serration design can substantially reduce the noise emissions of 5.2 dB at $\bar{h}$ = 2. Additionally, compared to single-wavelength serrations, an additional 1.47 dB noise reduction effect can be obtained by double-wavelength serrations under the appropriate design parameters. The surface pressure and phase distribution of different spanwise-varying leading edges indicate that the phase interference effect affected by source-radiated noise reduction is enhanced by this serration at the hills for serrations with a small curvature, and noise emission in the low-frequency band is more effectively suppressed. The sharper the serration is, the more conducive it is to a reduction in high-frequency noise. Nevertheless, the effectiveness of serrations is usually partially limited by the non-negligible trailing-edge self-noise. The sound source intensity of the root is decreased by the ogee-shaped serrations with a large curvature transition. A secondary noise reduction mechanism with a local source cut-off effect caused by nonlinearity is demonstrated.

## 1. Introduction

Organisms have developed unique characteristics throughout billions of years of evolution to adapt to the natural world. With the need for production and the development of science and technology, human beings gradually realized that the silent flight capacity of a flying owl is one of the important ways to open up new technologies, which provides a steady stream of inspiration for researchers. Hersh et al. explored the feasibility of utilizing leading-edge serrations to suppress aeroacoustics for the first time [1]. Distinguishingly, the silent flight capability of owls profits from the comb-like features of the leading edge and trailing edge, as well as downy upper surface features [2]. In recent years, a large number of studies have also demonstrated the effectiveness of leading-edge serrations in sound suppression in actual applications [3,4,5,6,7,8]. This research on leading-edge structures is mostly based on serrations with a single shape or wavelength, and it is difficult to give full play to the noise reduction potential of biomimicry. Therefore, this paper aims to explore the greater noise reduction capacity of biomimetic serration designs, employing a method for quickly predicting acoustics.

Noise generated by the strong interaction between the unsteady wakes and leading edge of downstream blades manifests as leading-edge noise in general [9]. This efficient noise radiation, regarded as the dominant noise source, forms in multi-row rotor systems. Moreover, leading-edge noise plays a crucial role in the noise emission of wind turbines on account of unsteady flow in sophisticated natural incoming flows. Noise, one of the most important design indicators, has been incorporated into the aerodynamic design of advanced wind turbines [10]. It is of prime importance in achieving economic benefits and of practical significance for decreasing the aerodynamic self-noise generated by wind turbines. Although the study of serrated leading-edge noise reduction has made great progress in experimental research and numerical simulation, critical noise reduction mechanisms that can only be speculated from experimental and numerical results can be more easily highlighted through analytical solutions. In particular, previous studies [8,11,12] demonstrate that varying leading-edge serrations in different flow environments exhibit different acoustic performance. Furthermore, if the scattering acoustic field of an arbitrary leading-edge geometry is determined, the noise reduction effect can be quickly predicted by an analytical model over a corresponding flow parameter range without repeating experiments or numerical simulation. Therefore, this analytical solution is equivalent to providing an effective theoretical method for quickly predicting the noise reduction ability of different leading-edge serrations in the corresponding flow state.

Flat plates have important theoretical reference value in studying acoustic mechanisms [13]. As an early representative study, Amiet [14] utilized the procedure of Schwarzschild and Landahl to obtain a closed-form approximate solution for the pressure and lift of the airfoil with the characteristic of a flat plate and infinite span. The far-field acoustic performance was confirmed to be related to the wave number spectral density of the vertical velocity fluctuations. In addition, if the wave number spectral density is extraordinarily precise, it is supposed to accurately predict the precise far field by Amiet’s theory and show good agreement with experiments [9]. To predict the noise emitted by airfoils with leading-edge serrations in a subsonic turbulent stream, Lyu et al. [15] expanded Amiet’s theoretical model and applied it to an airfoil with a serrated leading edge. Fourier expansions and Schwarzschild techniques were adopted to develop an analytical model. The purpose was to solve a set of coupled differential equations iteratively. Moreover, the far-field sound power spectral density was established by the statistics of incoming turbulent velocity. In light of this model, the destructive interference of scattered pressure is considered as the primary noise reduction mechanism. Recently, Huang et al. [16] proposed a theoretical model for exploring the scattering of sound waves generated from an arbitrary but periodical serrated flat edge in a uniform flow. Fourier series expansions and the Wiener–Hopf method were incorporated in this method to find the analytical solution. It was concluded that the cut-off of the scattered frequency caused the noise reduction. However, the complex details are hidden by the Fourier series expansions, as well as numerical Wiener–Hopf factorization. Since it is difficult to distinguish the final solution and determine when and where each term comes from, it has become a struggle to find a more essential noise reduction mechanism.

Additionally, in a preliminary study by Envia [17], alluding to the problem of an isolated finite-span swept airfoil interaction with convective gusts, Fourier transform methods and the Wiener–Hopf technique were employed to calculate an approximate solution. This was conducive to obtaining a closed-form expression of the acoustic far field and performing a parameter study. Subsequently, inspired by this research, a more concise analytical solution proposed by Ayton and Kim in 2018 promotes understanding of the noise reduction mechanism [18]. Taking analytical solutions corresponding to the Wiener–Hopf technique into consideration, the control equations and boundary conditions are converted through a series of variable transformations. As a result, any numerical steps during the solving of the far-field are avoided. The analytical model gives an insight into the aeroacoustics involved. This mathematical model is conducive to providing a research direction on aerodynamic noise mitigation for efficient design criteria for serrations.

Recent studies have shown that the optimization of different shapes of serrations have considerable potential for reducing aerodynamic noise. Lyu et al. [9] proposed that sharper serrations can acquire greater noise reduction levels in the high-frequency region. Compared with traditional serrations, an additional 7 dB noise reduction effect is achieved in the intermediate-frequency range, while there is no significant increase in the noise spectrum in other frequency bands. Wang et al. designed airfoils with serrated leading edges with different bionic elements. The iron-shaped leading-edge serrations were found to play a significant role in reducing leading-edge noise, with an additional 3.44 dB noise reduction compared with traditional serration [10]. Chaitanya et al. [19] proposed that double-wavelength serrations composed of the superposition of frequency components of different frequencies, amplitudes, and phases are sufficiently effective for specific frequency bands compared with single-wavelength serrations. For accommodating the intricate situations of practical applications, the double-wavelength characteristics and optimal noise reduction level of different serrations deserve further research to elucidate the broader noise reduction mechanisms.

Mathews’s work [20] in 2015 revealed that the optimum level of serrations is difficult to predict. We cannot acquire a type of serration that is sufficient to reduce noise under all parameters of any eddy current. The ideal state would be to achieve adaptive noise reduction in arbitrary incoming flows through varying serrations, which may be feasible in the future, but it is undoubtedly full of challenges for the time being. Enlightened by the above research, theoretical noise reduction research on double-wavelength serrations is conducted on account of the considerable noise reduction potential. This paper adopts the noise reduction theory of source control and radiation control of an analytical model as the starting point. Simultaneously, the effect of trailing-edge self-noise on high frequency is also taken into account. It is expected that this fundamental research can serve as an essential guide for clarifying how these serrations change acoustic performance and developing more acoustically effective serrations.

## 2. Analytical Formulation

The analytical method in this paper was proposed by Ayton and Chaitanya [21]. In this method, the linear inviscid equation is solved by the technique of the separation of variables and the Wiener–Hopf method. By integrating the solution of a single-frequency gust, the acoustic results of isotropic turbulence based on the single-value periodic spanwise-varying leading edge are obtained. The theory has been verified in relevant experiments [9]. It is worth noting that this method is highly effective in terms of saving calculation costs and exploring more essential noise reduction mechanisms. Once the flow parameters of any leading-edge geometry have been determined, repeated experiments and numerical simulation can be avoided to quickly obtain low-noise leading-edge shapes.

**Figure 1:**
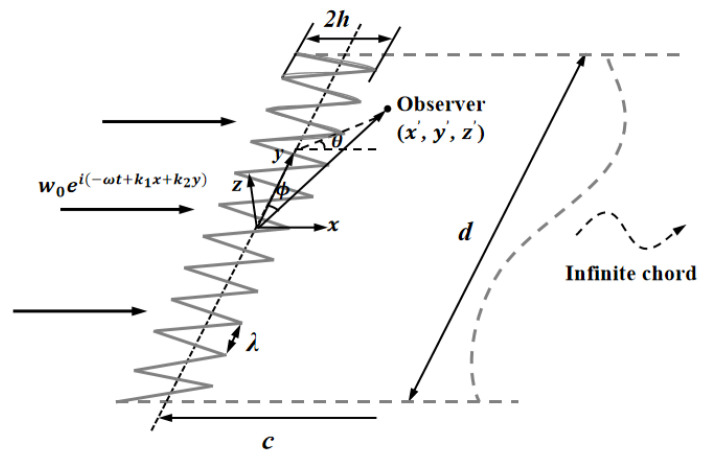
Schematic illustration of the leading-edge serrations.

As illustrated in Figure 1, a semi-infinite plate placed in a uniform incoming flow at a $0^{{}^{\circ}}$ attack angle is assumed to be the serrated airfoil, where *x* and *y* are the streamwise and spanwise directions, respectively. The *z* axis is parallel to the direction perpendicular to the airfoil plane [9,22]. Simultaneously, the serrated airfoils are supposed to be zero-thickness flat plates. Apart from that, the analytical model in this paper also requires meeting the subsequent conditions: (1) The gust is considered a two-dimensional spectrum $w_{o}e^{i(-\omega t+k_{1}x+k_{2}y)}$. The involved parameter *w_o_* is the gust amplitude of the upstream velocity, and *t* denotes the time. *k*_1_ and *k*_2_ represent the wave numbers in the streamwise and spanwise directions, respectively. (2) Taylor’s frozen hypothesis is considered in the incoming boundary layer. That is, the turbulence in the boundary layer is assumed to be frozen. (3) The investigated airfoil is periodic in the spanwise direction. The spanwise correlation length of the boundary layer turbulence is deemed to be much less than the wingspan [23].

Vertical velocity fluctuation is of great concern when the scattered potential mean flow is induced. This is closely related to the noise emission generated by the leading edge. Any continuous periodic leading-edge geometry F(y) with piecewise linear approximation characteristics is a single-valued linear function with a maximum value of 1 and a minimum value of −1. The geometric dimension is uniformly normalized by the wavelength of the serration. The spanwise serration is restricted to the smallest normalized period 1, owing to the fact that F(y) is periodic. As a consequence, the spanwise region is limited to the range of 0≤z≤1. The coordinate origin is distributed in the middle of the amplitude of serrations. The root-to-tip length is defined as h and the geometric profile is modeled as *hF*(*y*). The calculation procedures in light of the acoustic scattering prediction are derived from the following equations. The tip-to-root length of the serration is normalized by the wavelength and expressed as $\bar{h}$.

The Fourier transform is performed in the convective Helmholtz equation. Afterward, the Wiener–Hopf equation and non-orthogonal coordinate system transformation based on the above equation are combined for variable separation; then, the analytical solution is obtained. More detailed derivation processes can be found in reference [21]. The far-field sound power spectral density ψ at a specific monitoring position at a given frequency ω is given by the following:

(1) $$\psi \approx \frac{1}{\pi }\cos^{2}\frac{\theta }{2}\int_{-\infty }^{+\infty }{\left|\sum_{n=-\infty }^{\infty }\frac{\frac{k_{1}}{\beta^{2}}-w_{n}\mathrm{cos\theta}}{{\bar{k}}_{1}-w_{n}\mathrm{cos\theta}}\frac{1}{\sqrt{{\bar{k}}_{1}+w_{n}}}\frac{e^{iw_{n}r}}{\sqrt{r}}e^{i\chi_{n}y}E_{n}(-w_{n}\mathrm{cos\theta})\right|}^{2}\Phi^{\left(\infty \right)}\left(k_{1},k_{2}\right)dk_{2}.$$

where $\beta^{2}=1-M^{2};k=\omega /c_{o};k_{1}=k/M;$ $\bar{k}=k/\beta ;{\bar{k}}_{1}=k_{1}/c_{o}$; $w_{n}^{2}={\left(\delta M\right)}^{2}-\chi_{n}^{2},\chi_{n}=$ $\pm k_{2}+2n\pi ;$ $\delta =k_{1}/\beta$. M is the Mach number and $c_{o}$ refers to the local sound velocity.

The radiation integral equation $E_{n}$ associated with shape function F(η) is defined by

(2) $$E_{n}\left(-w_{n}cos\theta \right)=\int_{0}^{1}e^{i\left({\bar{k}}_{1}-w_{n}cos\theta \right)\bar{\Theta }F(\eta )}e^{-i2n\pi \eta }d\eta .$$

where $\bar{\Theta }=h/\beta$.

In Equation (1), $\phi^{\left(\infty \right)}(k_{1},k_{2})$ denotes the energy spectrum (Liepmann spectrum) of the upstream vertical fluctuation [24]. It is defined by

(3) $$\Phi^{\left(\infty \right)}\left(k_{1},k_{2}\right)=\frac{3\bar{u^{*2}}\Lambda^{*2}}{4\pi }\frac{\Lambda^{2}\left(k_{1}^{2}+k_{2}^{2}\right)}{{\left(1+\Lambda^{2}\left(k_{1}^{2}+k_{2}^{2}\right)\right)}^{\frac{5}{2}}},$$

where Λ is the turbulence integral length scale and $\Lambda^{*}\;is$ the turbulence integral length scale nondimensionalized by the wavelength. Here, Λ was set as 7.5 mm and the turbulent intensity $u^{*}$ was defined as 2.5%. $p^{*}$ represents dimensional pressure, which is defined as $\rho_{{}^{\circ}}^{*}U^{*2}p(r,\theta ,z)$. $\rho_{{}^{\circ}}^{*}$ is the airflow density (1.225 $kg\cdot m^{-3}$).

## 3. Comparison with Experiments

To verify the validity of this analytical model, the mathematical analytical results were compared with the experimental results [21]. In the experiment, flat plates were established to reduce manufacturing costs. The mean chord measuring 150 mm and span measuring 450 mm were taken to complete the parameter setting of the flat-plate airfoils. Also, the flat plates were composed of 1 mm thick metallic sheets superimposed with 2 mm thick serrated flat plates embedded in the middle. Serrations with tip-to-root ratios $\bar{h}$ of 1, 2, and 4 were implemented to analyze the acoustic sensitivity of the sound source in this study. More details of the experimental equipment are elaborated in Ref. [25]. The dimensions concerning the open jet test facility in an anechoic chamber were 8 m × 8 m × 8 m. The airflow was provided by a centrifugal fan installed on the ceiling of the anechoic chamber and driven by a variable-speed motor. Moreover, to generate a quiet, uniform, and low-turbulence flow, a number of grids and honeycomb structures located in the silencing channel were set up. Moreover, the microphones of the sound source were distributed at the position on the circular arc with a radius of 1.2 m, away from the leading edge of the airfoil. The array of these receiving points was covered over the angle range relative to the downstream jet axis of 40° to 140°. The sampling frequency was 50 KHz and the window size was 1024 data points. Hence, the corresponding frequency resolution was 48.83 Hz. Here, the velocity spectrum with a mean flow velocity was extracted and compared with the analytical velocity spectrum at the corresponding flow rate.

**Figure 2:**
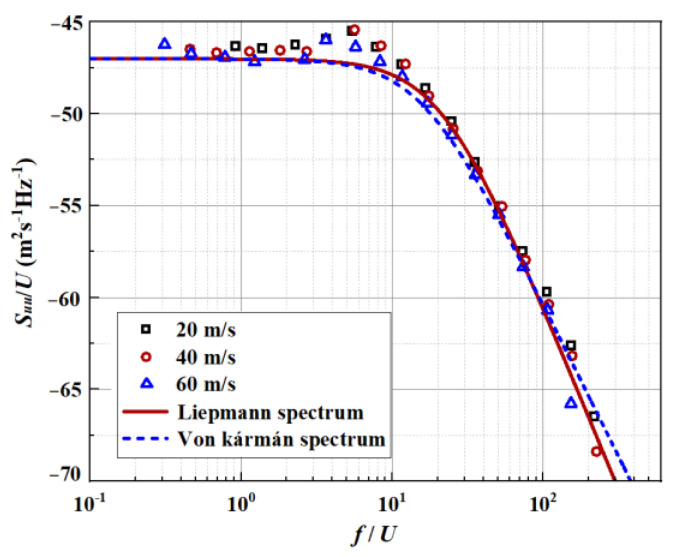
Comparison between the measured axial velocity spectra and theoretical spectra.

The one-dimensional streamwise velocity spectrum was estimated by the von Kármán spectrum in Equations (4) and (5). Apart from that, the Liepmann spectrum illustrated in Equation (6) is also discussed here. The isotropic turbulence assumption was adopted here. And the homogeneous turbulence spectrum model as $S_{11}(k_{1})=\phi_{11}(k_{1})/U_{1}$ was estimated by Taylor’s hypothesis [26]. Notably, division by mean velocity ($U_{1}$ = 60 m/s) is necessary to ensure that $S_{11}(k_{1})$ integrates into the mean square velocity fluctuation. Figure 2 compares the streamwise velocity spectrum produced by boundary layer turbulence estimated by the Liepmann and von Kármán spectra, respectively. Except for some nonessential discrepancies in the mid-frequency region, the estimates of both spectra show good consistency with the velocity spectrum measured in the experiment. The Liepmann spectrum, more fitting for this experiment, was employed in this paper.

(4) $$\phi_{11}\left(k_{1}\right)=\frac{1}{\sqrt{\pi }}\frac{\Gamma \left(5/6\right)}{\Gamma \left(1/3\right)}\frac{\bar{u^{2}}}{k_{e}}\frac{1}{{\left[1+{\left(k_{1}/k_{e}\right)}^{2}\right]}^{5/6}},$$

(5) $$k_{e}=\frac{\sqrt{\pi }}{\Lambda }\frac{\Gamma \left(5/6\right)}{\Gamma \left(1/3\right)},$$

(6) $$\overset{´}{\phi_{11}}\left(k_{1}\right)=\frac{\bar{u^{2}}\Lambda }{\pi }\frac{1}{1+{\left(k_{1}\Lambda \right)}^{2}}.$$

On account of ignoring the trailing-edge self-noise, Lyu’s analytical model [22] differs greatly from the experimental results at the high-frequency band, especially for serrations with a considerable tip-to-root ratio. However, the effect of self-noise emitted by the trailing edge is inevitable in the experimental setup. Narayanan et al. [26] mentioned that trailing-edge self-noise plays a more dominant role over leading-edge self-noise in some specific frequency ranges. The acoustic performance of leading-edge serrations has declined to some extent due to the emergence of trailing-edge self-noise. Afterward, Ayton’s study took into account the contribution of trailing-edge self-noise, meaning that the results of the analytical model are in good agreement with the experiment. Since the influence range of self-noise is within the frequency band in question, the effect of trailing-edge self-noise must be considered when performing the acoustic evaluation.

The sound pressure level contributed by the trailing-edge self-noise ${SPL}_{TE}$ is acquired by the method of Brooks, Pope, and Marcolini [27]. The fundamental theory established by Amiet does not serve as a very practical prediction tool in evaluating the relationship between the trailing edge and the boundary layer pressure fluctuations. Based on that, Brooks, Pope, and Marcolini (BPM) measured the self-noise of airfoils over a range of conditions. Furthermore, some empirical correlations with the measurement data were established. The BPM method has been the primary choice for baseline comparison for some sophisticated noise prediction schemes since it was proposed. It is worthwhile to note that the measured sound spectrum of the BPM method is derived from Amiet and Ffowcs-Williams and Hall [28]. Then, the normalized spectral forms and correlations of boundary layer scaling variables can be obtained precisely under a specified condition.

The total TBL-TE (turbulent boundary layer–trailing edge) is defined as

(7) $${SPL}_{TE}=10log\left(10^{\frac{{SPL}_{\alpha }}{10}}+10^{\frac{{SPL}_{s}}{10}}+10^{\frac{{SPL}_{p}}{10}}\right).$$

where ${SPL}_{p}$ and ${SPL}_{s}$ denote the noise spectral functions caused by the pressure and suction side, respectively. ${SPL}_{\alpha }$ denotes the angle-dependent noise spectral functions. The definitions of them are as follows:

(8) $${SPL}_{p}=10\log\left(\frac{\delta_{p}^{*}M^{5}L{\bar{D}}_{h}}{r_{e}^{2}}\right)+A\left(\frac{{St}_{p}}{{St}_{1}}\right)+\left(K_{1}-3\right)+\Delta K_{1},$$

(9) $${SPL}_{s}=10\log\left(\frac{\delta_{s}^{*}M^{5}L{\bar{D}}_{h}}{r_{e}^{2}}\right)+A\left(\frac{{St}_{s}}{{St}_{1}}\right)+\left(K_{1}-3\right),$$

(10) $${SPL}_{\alpha }=10\log\left(\frac{\delta_{s}^{*}M^{5}L{\bar{D}}_{h}}{r_{e}^{2}}\right)+B\left(\frac{{St}_{s}}{{St}_{2}}\right)+K_{2}.$$

The Strouhal definitions of ${St}_{p}$ and ${St}_{s}$ are

(11) $${St}_{p}=\frac{f\delta_{p}^{*}}{U},{St}_{s}=\frac{f\delta_{s}^{*}}{U},$$

(12) $${St}_{1}=0.02M^{-0.6},$$

(13) $${St}_{2}={St}_{1}\times \left\{\begin{array}{c} 1\;\;\;\;\;\;\;\;\;\;\;\;\;\;\;\;\;\;\;\;\;\;\;\;\;\;\;\;\;\;\;\;\;\;\;\;\;\left(\alpha_{*}<1.33^{{}^{\circ}}\right)\\ 10^{0.0054{\left(\alpha_{*}-1.33\right)}^{2}}\left(1.33^{{}^{\circ}}<\alpha_{*}<12.5^{{}^{\circ}}\right).\\ 4.72\;\;\;\;\;\;\;\;\;\;\;\;\;\;\;\;\;\;\;\;\;\;\;\;\;\;\;\;\;\;\;\left(\alpha_{*}>12.5^{{}^{\circ}}\right) \end{array}\right.$$

where $\alpha_{*}$ is the attack angle. $\delta_{p}^{*}$, $\delta_{s}^{*}$, and $\delta_{\alpha }^{*}$ are the boundary layer displacement thicknesses caused by the pressure side, the suction side, and the angle of attack, respectively; *L* is the span length (*L* = 450 mm); and ${\bar{D}}_{h}$ is the directivity function. More details on the definition of related parameters are elaborated in Ref. [27].

The sound pressure level is described as

(14) $$SPL=10{log}_{10}\left(\int_{-\infty }^{\infty }\frac{{\left|p^{*}\right|}^{2}\phi^{\left(\infty \right)}\left(k_{1},k_{2}\right)dk_{2}}{p_{ref}^{2}}\right)+{SPL}_{TE},$$

where $p_{ref}=2\times 10^{-5}\;Pa.$

We considered the most sensitive range of human hearing: 0~10,000 Hz. The Kutta condition is that the airflow on the upper and lower surfaces of the airfoil gathers at the trailing edge of the airfoil, and then generates velocity circulation to provide lift for the airfoil. However, this theory is based on a semi-infinite flat plate, so the Kutta condition cannot be applied here. Therefore, the Kutta condition affected by the Coanda effect is not considered in this analytic model, which impacts noise prediction within the frequency range of f≲500 Hz [21]. Accordingly, the frequency range of 500 Hz to 10,000 Hz was taken for comparison between analytical solutions and experiments. It should be noted that there is an obvious contrast between the analytical solution in the low-frequency range and the experimental result. Previous studies [22,26] have shown that low-frequency noise measured in the far field is dominated by jet noise, so noise prediction in the low-frequency region was not our focus. Moreover, the analytical model is in good agreement with the experimental results when the frequency *f* is greater than 2000 Hz.

**Figure 3:**
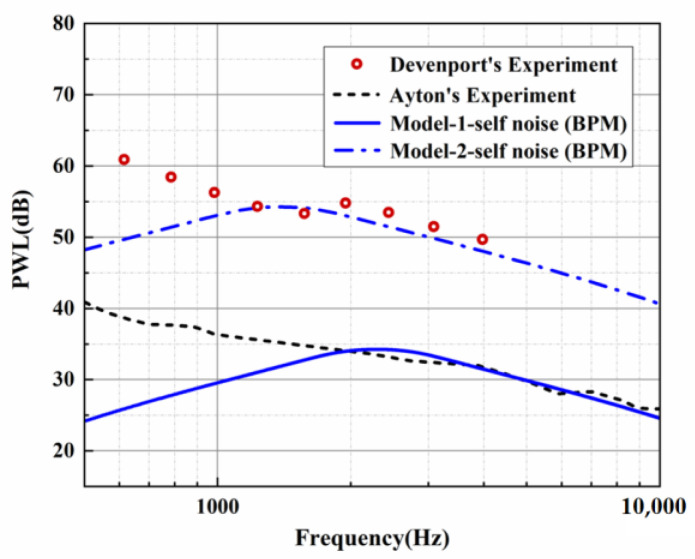
SPL comparison for analytical and experimental results of trailing-edge self-noise.

**Figure 4:**
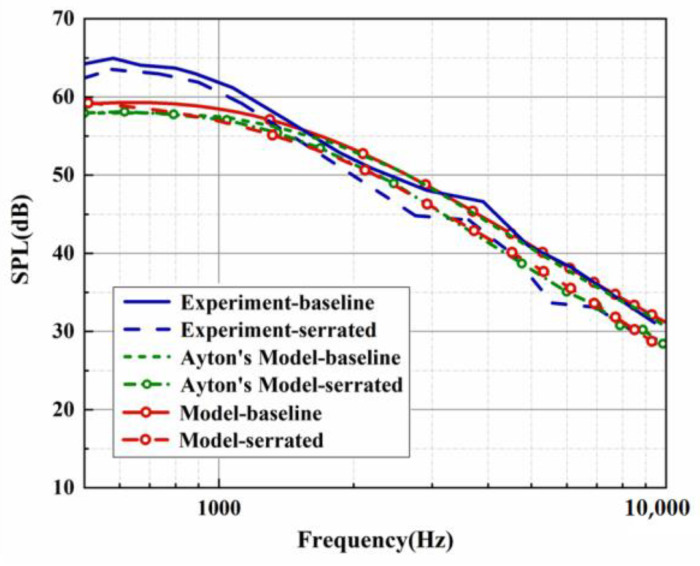
SPL generated by the analytical results in view of self-noise and experimental results at $\bar{h}=0.5$.

**Figure 5:**
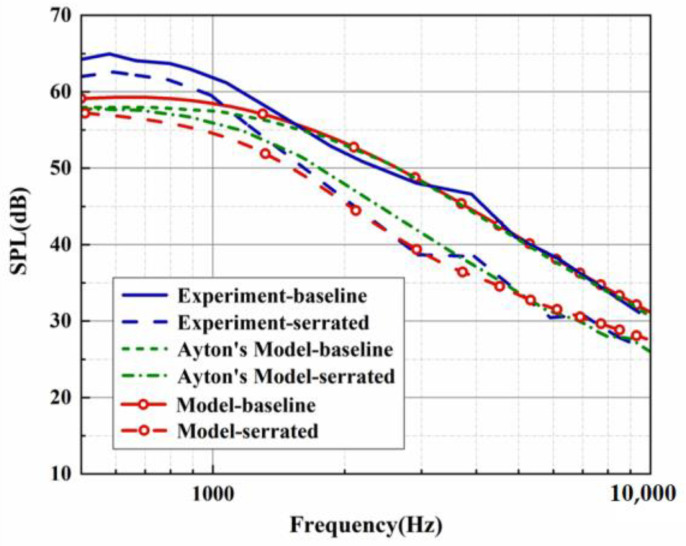
SPL generated by the analytical results in view of self-noise and experimental results at $\bar{h}=1$.

**Figure 6:**
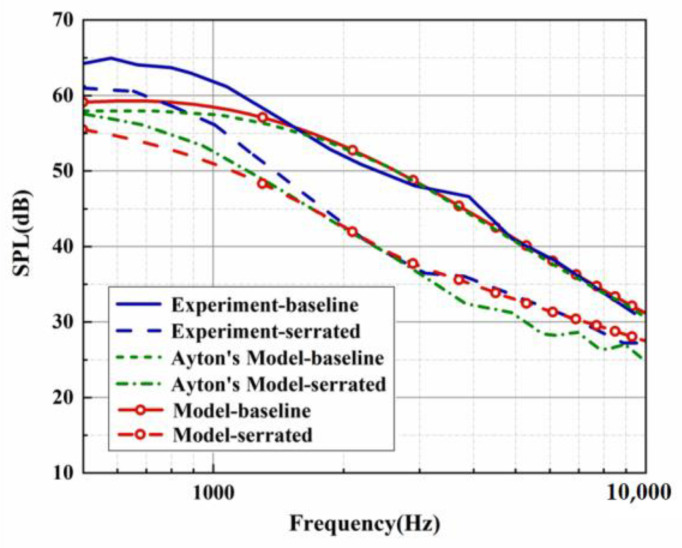
SPL generated by the analytical results in view of self-noise and experimental results at $\bar{h}=2$.

Both the predicted and experimental measurements exhibited similar oscillation behavior. Filtering was performed to more clearly compare the noise reduction performance of each serration. However, in the evaluation of the overall sound pressure level (OASPL), broadband noise, and the directionality of different azimuths, the data before the filtering were adopted to capture more local details of the sound pressure spectrum. The BPM model was verified by a standard NACA0012 airfoil under a mean flow state. The trailing-edge noise spectrum for a tripped NACA0012 at an angle of attack of zero evaluated by the BPM model is depicted in Figure 3. The key parameters are listed as follows. This experiment was performed at a flow speed of 55.5 m/s, a chord length of 0.23 m, and a span of 1.22 m. The observation angle was located 3 m above the mid-span of the trailing edge. With these key parameters, we can estimate the trailing-edge noise of model 2 (NACA0012 airfoil) at the corresponding operation condition. Except for the lower-frequency band, the sound pressure level of the higher-frequency band shows good consistency with the experimental results measured by Devenport et al. [29]. The effectiveness of the BPM model was confirmed. Furthermore, the trailing-edge noise of the investigated subject corresponding to model 1 (flat plate) in this study is also exhibited in Figure 4. Similar to model 2, the analytical results and Ayton’s experiment are in good agreement for the SPL distribution in the frequency band over 2000 Hz. As described in Figure 4, Figure 5 and Figure 6, when the trailing-edge self-noise is incorporated in the overall sound pressure level, taking Ayton’s analytical model as a reference, it can be seen that the analytical models of smooth airfoils and serrated airfoils have a high degree of coincidence with the experiment in the medium- and high-frequency regions.

## 4. Acoustic Performance of Single-Wavelength Serrations

**Figure 7:**
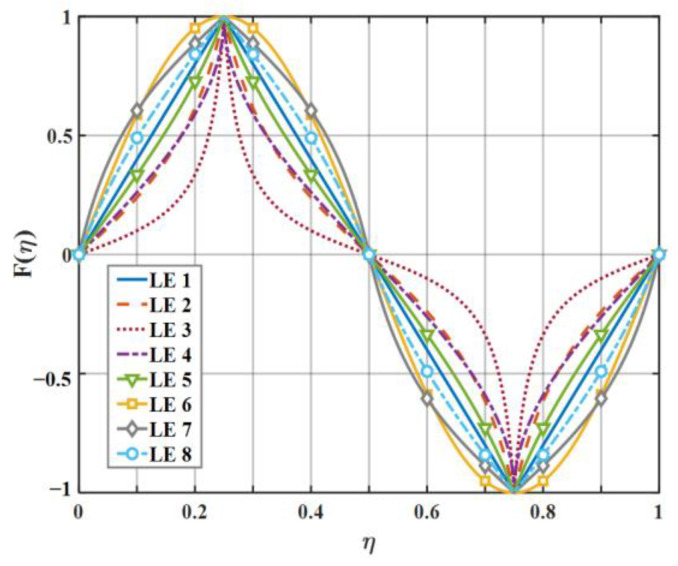
The serration profiles of diverse leading edges.

For the sake of clarifying the noise reduction mechanism and noise reduction effect of diverse serrations as clearly as possible, eight kinds of serrations, as exhibited in Figure 7, were initially picked to evaluate the sound pressure level in this paper. Each representative serration has various laws of curvature variation within the interval of each segment function. The mathematical expression related to LE 1–3 (leading-edge serrations 1–3) is described in Equation (16), where the b-values of LE 1, LE 2, and LE 3 are close to 0, 1.4π, and 1.8π, respectively. Similarly, the shape of LE 4–5 is controlled by Equation (17), where the b-values of LE 4 and LE 5 are 4 and 3.5, respectively. Relatedly, when the b value of LE 1 is close to 0, it represents the traditional trailing-edge serrations, and the remaining four b-values of LE 2–5 represent ogee-shaped serrations with different curvatures, respectively. The sinusoidal serrations depicted as LE 5 are modeled as Equation (18). Moreover, the iron-shaped serrations represented by LE 7–8 are expressed as Equation (19), where the b-values of LE 7 and LE 8 are 1.4π and π, respectively. What is noteworthy is that different b-values control the sharpness of different curvature shapes.

(15) $$F\left(\eta \right)=\left\{\begin{array}{c} \frac{1}{\tan\left(\frac{b}{4}\right)}\tan\left(b\eta \right),0\leq \eta \leq \frac{1}{4},\;\;\;\;\;\;\;\;\;\;\;\;\\ \frac{1}{\tan\left(\frac{b}{4}\right)}\tan(b(-\eta +\frac{1}{2})),\frac{1}{4}\leq \eta \leq \frac{3}{4},\\ \frac{1}{\tan\left(\frac{b}{4}\right)}\tan\left(b\left(\eta -1\right)\right),\frac{3}{4}\leq \eta \leq 1.\;\; \end{array}\right.$$

(16) $$F\left(\eta \right)=\left\{\begin{array}{c} \frac{1}{\arcsin\left(\frac{b}{4}\right)}\arcsin\left(b\eta \right),0\leq \eta \leq \frac{1}{4},\;\;\;\;\;\;\;\;\;\;\;\;\;\\ \frac{1}{\arcsin\left(\frac{b}{4}\right)}\arcsin(b(-\eta +\frac{1}{2})),\frac{1}{4}\leq \eta \leq \frac{3}{4},\\ \frac{1}{\arcsin\left(\frac{b}{4}\right)}\arcsin\left(b\left(\eta -1\right)\right),\frac{3}{4}\leq \eta \leq 1,\;\;\; \end{array}\right.$$

(17) $$F\left(\eta \right)=\sin\left(2\pi \eta \right),0\leq \eta \leq 1.$$

(18) $$F\left(\eta \right)=\left\{\begin{array}{c} \frac{1}{\tan\left(\frac{b}{4}\right)}\tan\left(b\left(\eta -0.25\right)\right)+1,0\leq \eta \leq \frac{1}{4},\;\;\;\;\;\;\;\;\;\;\;\;\;\;\;\;\;\;\\ \frac{1}{\tan\left(\frac{b}{4}\right)}\tan(b(-(\eta +0.25)+\frac{1}{2}))+1,\frac{1}{4}\leq \eta \leq \frac{1}{2},\;\;\;\;\;\;\\ \frac{1}{\tan\left(\frac{b}{4}\right)}\tan\left(b\left(-\left(\eta -0.25\right)+\frac{1}{2}\right)\right)-1,\frac{1}{2}\leq \eta \leq \frac{3}{4}.\;\\ \frac{1}{\tan\left(\frac{b}{4}\right)}\tan\left(b\left(\eta -0.75\right)\right)-1,\frac{3}{4}\leq \eta \leq 1.\;\;\;\;\;\;\;\;\;\;\;\;\;\;\;\;\; \end{array}\right.$$

**Figure 8:**
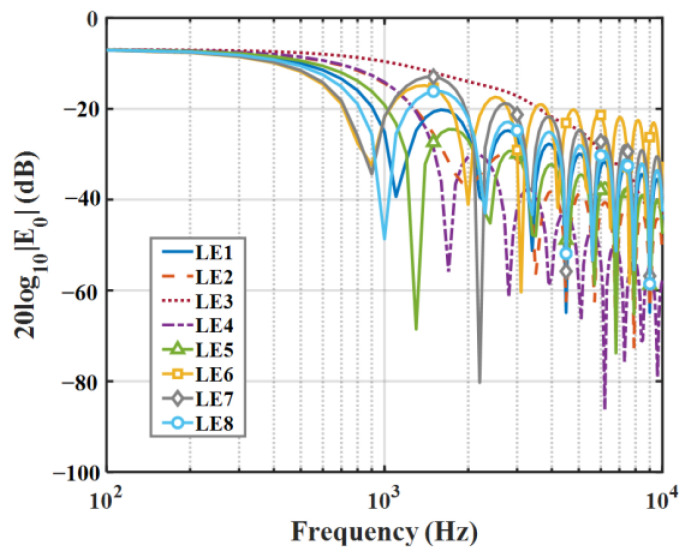
Comparison of decay rates of $\left|E_{0}\right|$ for different leading-edge serrations.

**Figure 9:**
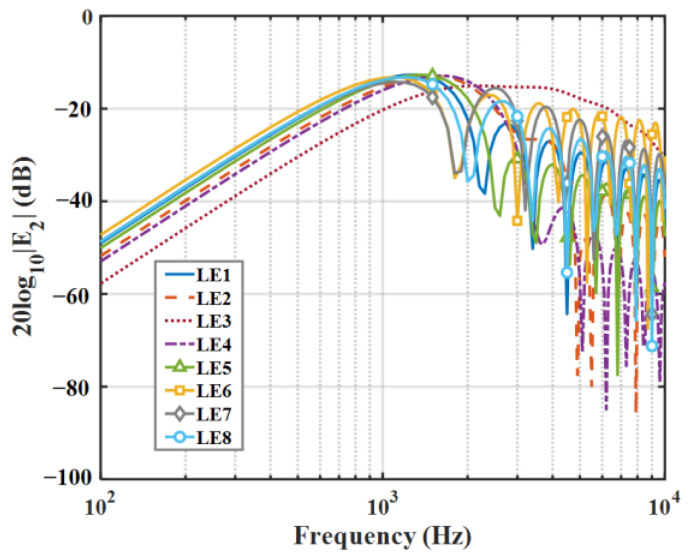
Comparison of decay rates of $\left|E_{2}\right|$ for different leading-edge serrations.

The $10{log}_{10}{\left|E_{n}\right|}^{2}$ distributions for the 0-order and 2-order modes of eight kinds of serrations are shown in Figure 8 and Figure 9. In general, not all modes contributed equally to the response, and higher-order modes had less effect on low frequencies. Therefore, modal truncation was performed, and higher-order modes were discarded. In this way, the matrix orders of the frequency response function were greatly reduced, which greatly reduced the workload. Concerning this serrated model, only the finite-order modes need to be considered, which is related to the degree of freedom of the system. In this system, the 0th-order mode is dominant, which is related to the degrees of freedom of this system. Overall, as the modal order increases, $\left|{E_{n}}^{2}\right|$ in the low-frequency region is dissipated partially, and the difference between different serrations moves toward the high-frequency region.

**Figure 10:**
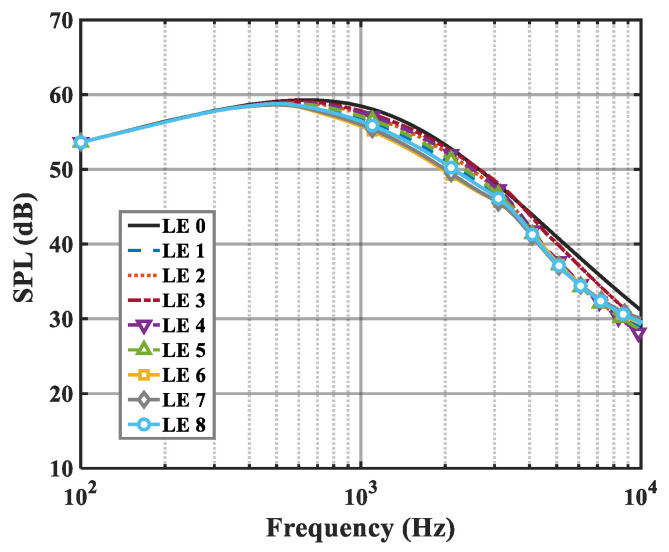
SPL spectra of different leading-edge serrations at $\bar{h}=0.5$.

**Figure 11:**
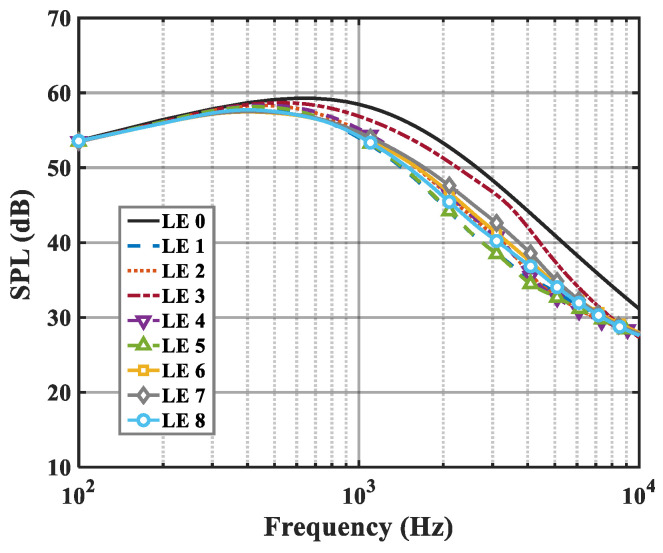
SPL spectra of different leading-edge serrations at $\bar{h}=1$.

**Figure 12:**
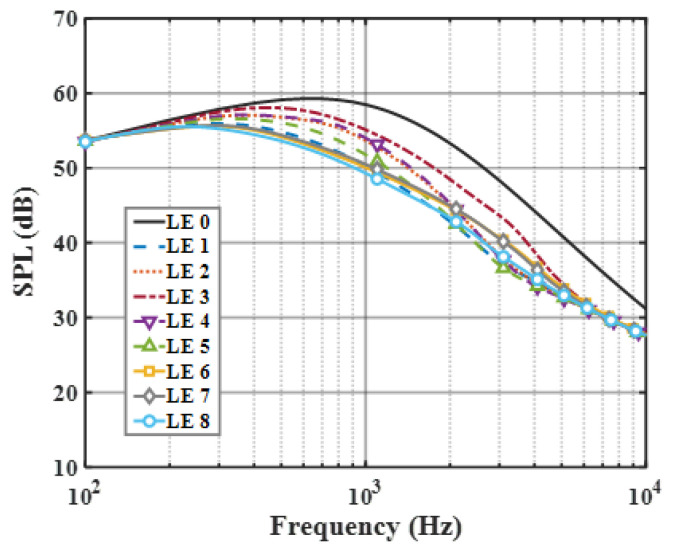
SPL spectra of different leading-edge serrations at $\bar{h}=2$.

**Figure 13:**
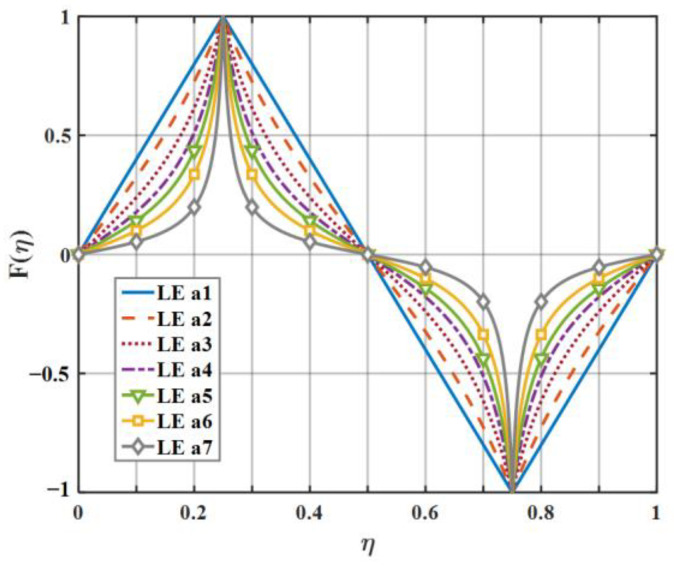
The serration profiles of the diverse leading edge with different values of b in Equation (16).

**Figure 14:**
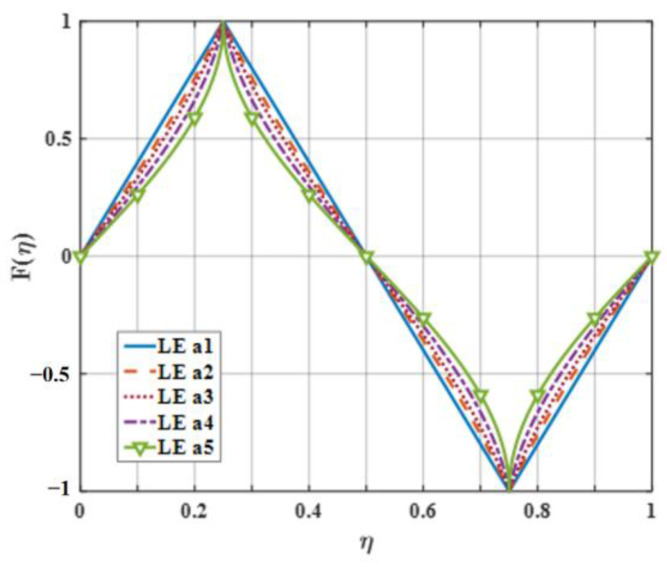
The serration profiles of the diverse leading edge with different values of b in Equation (17).

**Figure 15:**
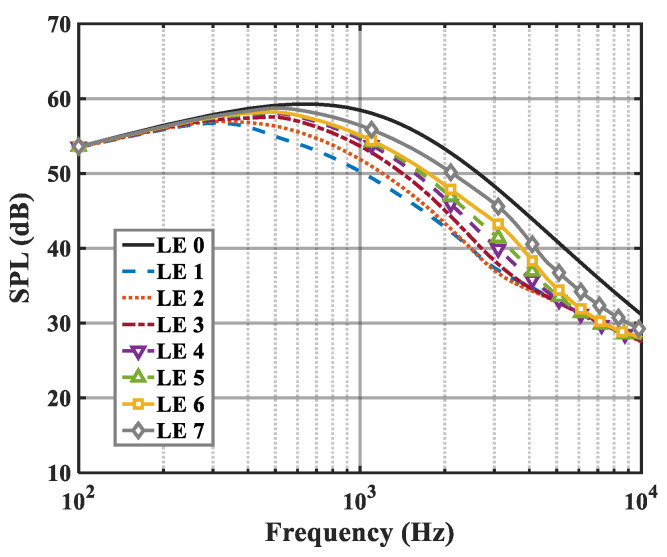
SPL spectra of leading-edge serrations with different b values in Equation (16) at $\bar{h}=2$.

**Figure 16:**
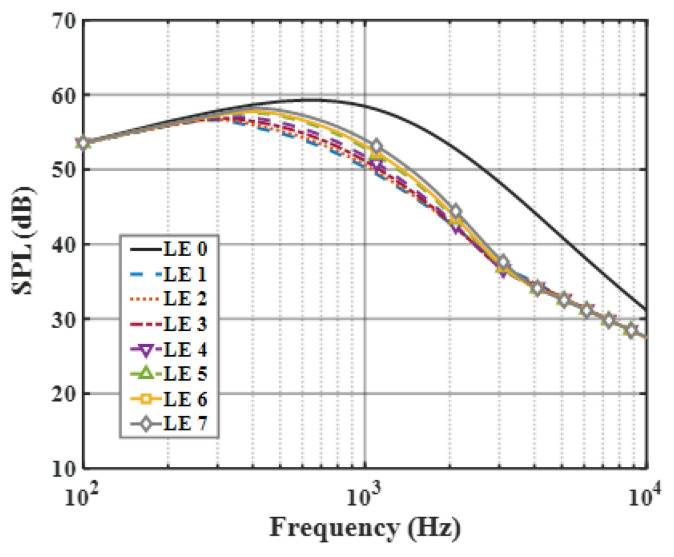
SPL spectra of leading-edge serrations with different b values in Equation (17) at $\bar{h}=2$.

On the basis of the experimental results, the finite modal numbers corresponding to the studied serrations with different root-to-tip ratios were obtained, as well as the SPL distribution in Figure 10, Figure 11 and Figure 12. It is worth noting that the LE 2, LE 4, and LE 5 with ogee shapes performed well at $\bar{h}=2$ in sound suppression. Thus, the serrations in Figure 13 and Figure 14 based on the tan and arcsine functions were carefully studied as two representatives. LE a1–a7 based on different b-values (b = 0, π, 1.4π, 1.6π, 1.7π, 1.8π, 1.9π) in Equation (16) and LE b1–b7 based on different b-values (a = 0, 2, 3, 3.5, 3.9, 3.95, 4) in Equation (17) were investigated in detail. As shown in Figure 15 and Figure 16, the overall SPL distribution of different b-value arcsine functions performs better than tan functions based on different b-values. This is related to the characteristics of the arcsine function, placing the b-values in the range of more effective noise reduction.

## 5. Acoustic Sensitivity of Double-Wavelength Serrations

The double-wavelength serrations proposed by Chaitanya et al. [19] are arranged in a manner in which adjacent roots are separated in the flow direction. In addition, the adjacent root distance is required to be close enough to the turbulence integral length scale. This double-wavelength serrated design based on phase interference provides inspiration for noise mitigation. Nevertheless, the noise reduction capabilities and influence mechanism of double-wavelength serrations composed of different amplitudes and shapes are still unclear, which is worth investigating further.

**Figure 17:**
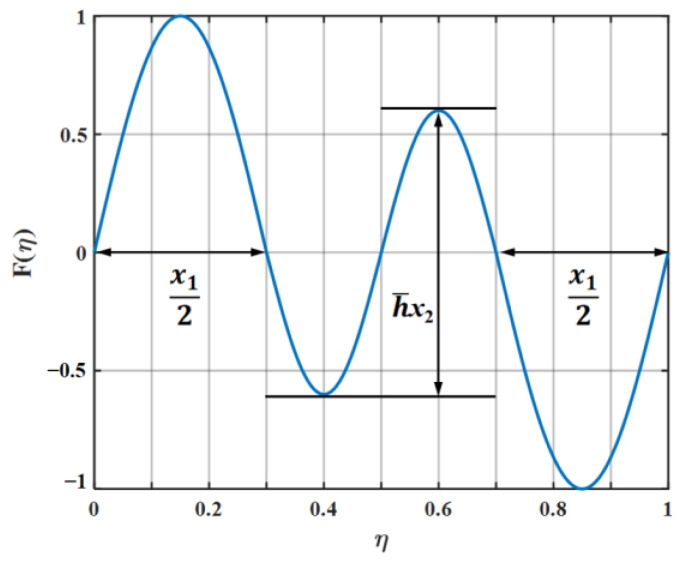
Schematic diagram of a double-wavelength serration.

Four representative geometries of serrated, ogee-shaped, sinusoidal, and iron-shaped serrations with different curvatures were employed to explore the aeroacoustics of double-wavelength serrations. The parameter settings for the double-wavelength serrations are depicted in Figure 17. A comprehensive trial design of five factors and four levels was performed, as shown in Table 1. Taking serrations of $\bar{h}=1$ as an example, the wavelengths of the double-wavelength serrations were all fixed at 25 mm. All length dimensions were normalized by wavelength. If one of the wavelengths of the double-wavelength serrations was determined, the other was also determined. After controlling partial variables, a comprehensive trial design of two factors (B, C) and four levels was formed when factor A remained stable, as demonstrated in Table 2.

Table 3, related to the trial results, summarizes the ranges of impact factors for different serrations. $R_{\bar{h}=0.5}$, $R_{\bar{h}=1}$, and $R_{\bar{h}=2}$ denote the range of three factors corresponding to shape factor A, wavelength B, and amplitude C. Aiming at $\bar{h}=2$, it was concluded that A was the factor of maximal impact and B was the factor of minimal impact. This implies that the variation in shape factor had the greatest influence on the sound pressure level at $\bar{h}=2$, followed by the amplitude, and the wavelength had the least influence on it. Similarly, the order conclusions of the factors’ sensitivity to other operation conditions were also derived.

(19) $$K_{Bi}=\frac{1}{4}(\sum_{n=1}^{n=4}I_{n+4(i-1)}).$$

(20) $$K_{ci}=\frac{1}{4}(\sum_{n=i}^{n=i+3}I_{i+4(n-i)}).$$

(21) $$R_{\bar{h}=1}=max\left(K_{ij}\right)-min\left(K_{ij}\right),\;$$

where $K_{ij}$ represents the arithmetic average value of all indicators of the factor i at the level of j. And $K_{Ci}$ is the arithmetic mean of all indicators for the factor C at the level i. $R_{\bar{h}=1}$ denotes the range of all calculated results of the factor i at the level j in the case of $\bar{h}=1$.

Affected by a complicated phase-interference mechanism, not every trial result of double-wavelength serration is superior to the single wavelength serration. So, we propose a comprehensive assessment index (SPL_CA_), which is defined as follows:

(22) $${SPL}_{CA}=\frac{\log_{0.5}\left({(n}_{{}^{\circ}}+0.5)/n\right)}{n\left|\log_{0.5}\left({(n}_{{}^{\circ}}+0.5)/n\right)\right|}\sum_{i=1}^{n}\left|SPL-{SPL}_{\lambda_{i}}\right|,n\geq 2,n_{o}\geq 1.$$

Taking $\bar{h}=1$ as an example, ${SPL}_{\lambda_{i}}$ is the sound pressure level at the wavelength of $\lambda_{i}$ (i=1,2) and the amplitude of $\bar{h}=1$. *n_o_* denotes the number of negative values that occurred for $SPL-{SPL}_{\lambda_{i}}$. *n* is the type of investigated wavelength contained in a period. Moreover, a negative value of ${SPL}_{CA}$ indicates that the noise reduction capacity of the double-wavelength serrations is superior to any of the involved single wavelengths of $\lambda_{i}$.

The shape factor, wavelength, and amplitude are the dominant factors affecting the sound pressure level, respectively. For different serrations, the effects of amplitude and wavelength exhibit different laws under the influence of different root-to-tip ratios. The shape factor always dominates regardless of the change in the $\bar{h}$-value. In short, the optimization of the shape function has considerable potential for the suppression of the overall sound pressure level. The influence of amplitude on the overall sound pressure level gradually enhances with the increase in $\bar{h}$. The variable *f*, related to the geometric shape, is introduced here. The variable *f* is employed to evaluate the effect of different geometric shapes on aerodynamic noise, which is defined as follows:

(23) $$f=A-A_{\circ }=\int_{0}^{1}F\left(\eta \right)d\eta -\int_{0}^{1}F_{\circ }\left(\eta \right)d\eta ,$$

where $A_{\circ }$ represents the areas enclosed by traditional serrations and the horizontal axis, and A is the area surrounded by random serrations and the horizontal axis. And, presumably, if f>0, it implies that the noise reduction in the low-frequency region is better than the noise reduction effect in the high-frequency region, and if f<0, the results obtained are simply the opposite.

**Figure 18:**
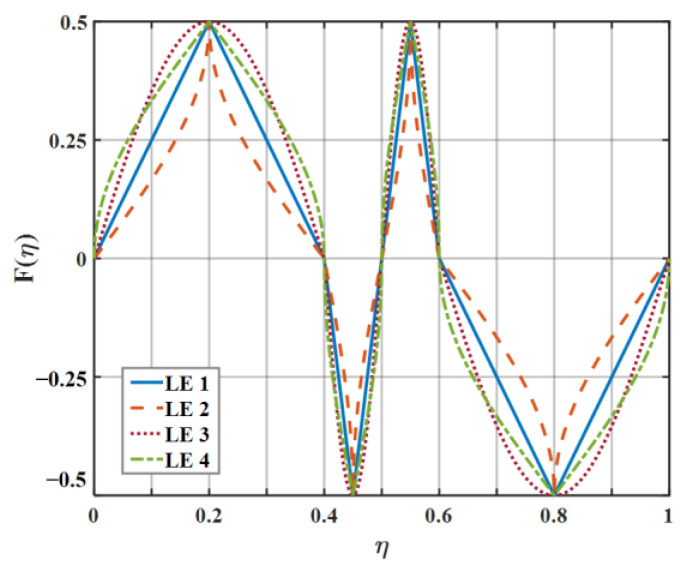
The serration profiles of leading edges with minimum test value (SPL) at $\bar{h}=0.5$.

**Figure 19:**
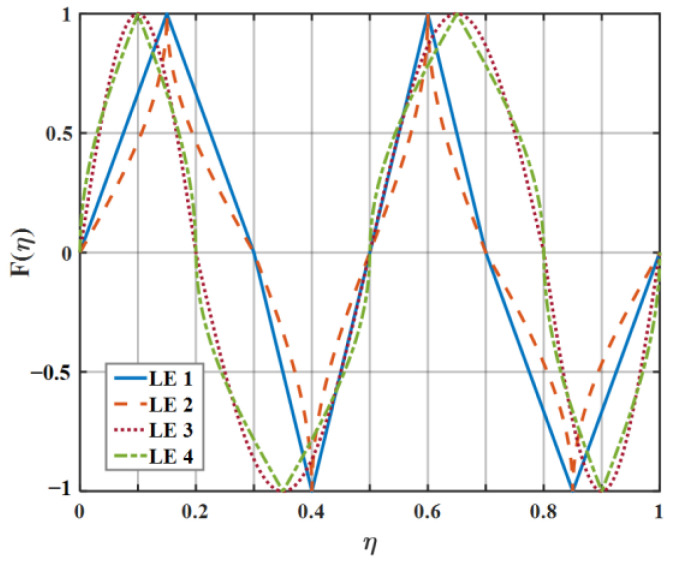
The serration profiles of lading edges with minimum test value (SPL) at $\bar{h}=1$.

**Figure 20:**
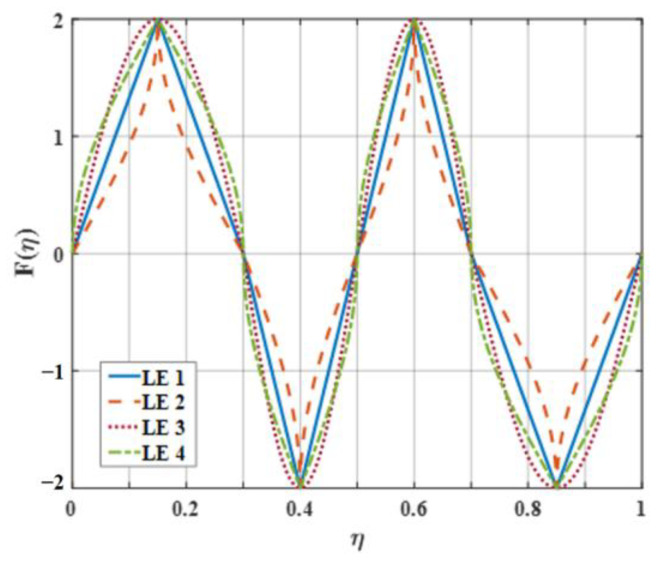
The serration profiles of leading edges with minimum test value (SPL) at $\bar{h}=2$.

**Figure 21:**
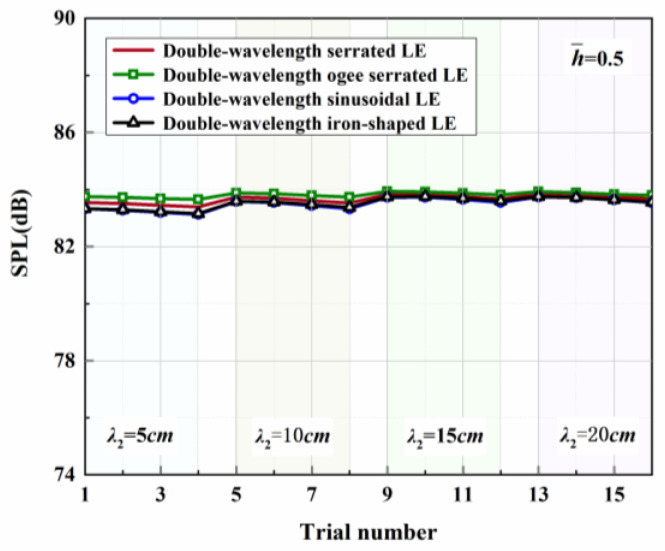
OASPLs of representative serrations with double wavelengths at $\bar{h}=0.5$.

**Figure 22:**
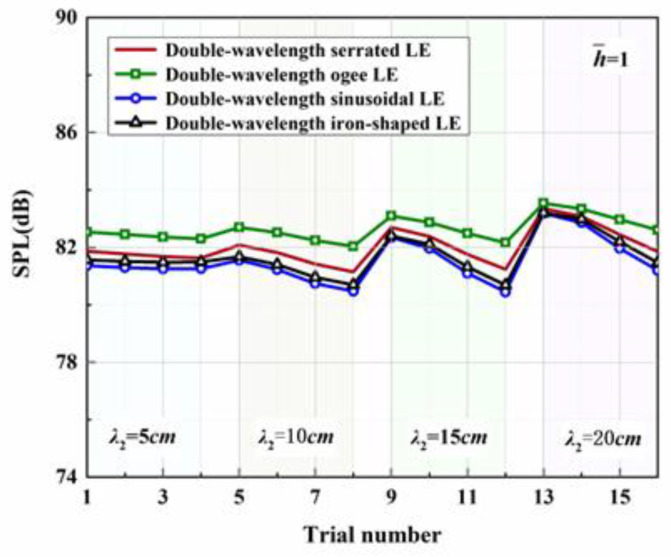
OASPLs of representative serrations with double wavelengths at $\bar{h}=1$.

**Figure 23:**
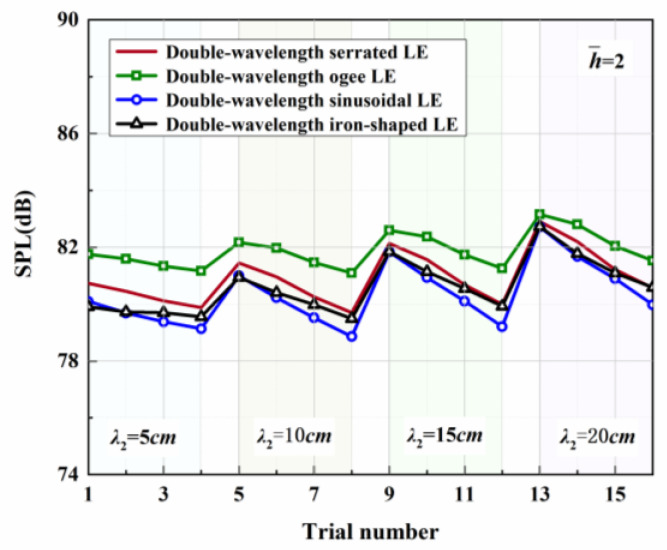
OASPLs of representative serrations with double wavelengths at $\bar{h}=2$.

**Figure 24:**
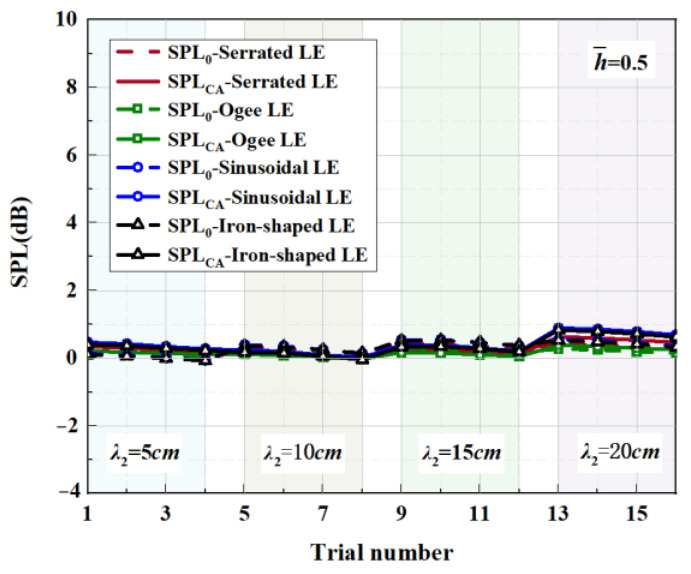
SPL_CA_ and SPL_0_ distributions of representative serrations with double wavelengths at $\bar{h}=$ 0.5.

**Figure 25:**
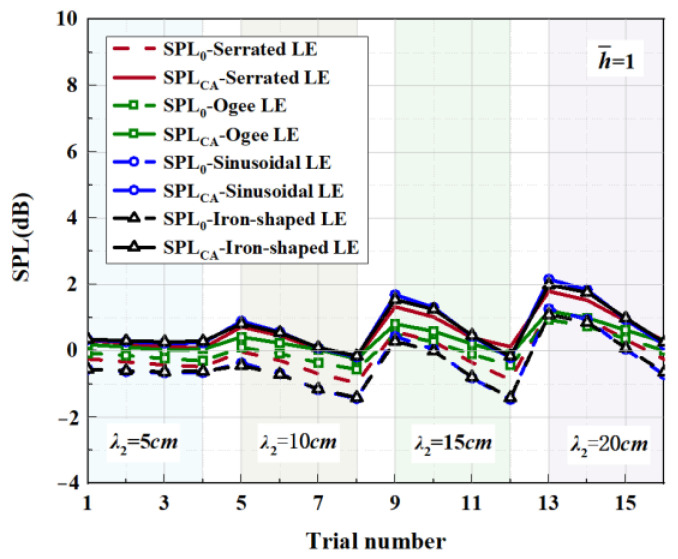
SPL_CA_ and SPL_0_ distributions of representative serrations with double wavelengths at $\bar{h}=$ 1.

**Figure 26:**
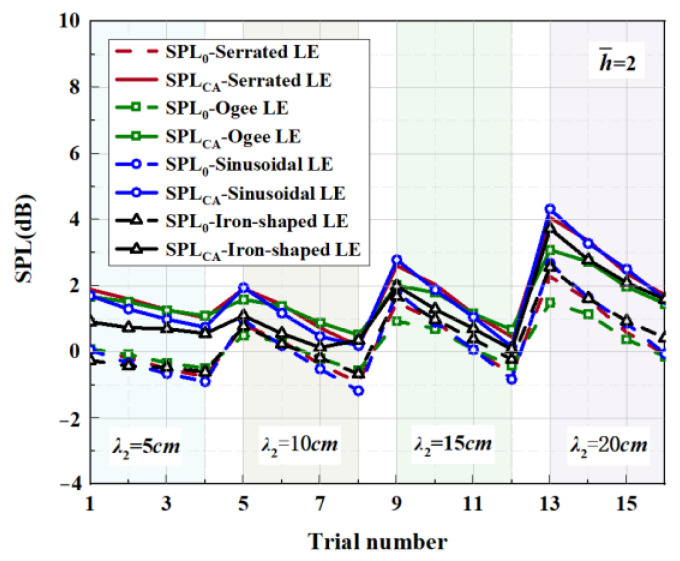
SPL_CA_ and SPL_0_ distributions of representative serrations with double wavelengths at $\bar{h}=$2.

The serration profiles of double-wavelength serrations with minimum test values are shown in Figure 18, Figure 19 and Figure 20. LE1–LE4 denote the serrated, ogee-shaped, sinusoidal, and iron-shaped serrations, respectively. The OASPLs related to different LE serrations are exhibited in Figure 21, Figure 22, Figure 23, Figure 24, Figure 25 and Figure 26. Similar to Figure 10, Figure 11 and Figure 12, smaller sound pressure levels can be achieved at a larger tip-to-root ratio. That is to say, sharper serrations are more likely to suppress aerodynamic noise, which is consistent with the conclusions reached by early researchers [9]. When the amplitude corresponding to $\lambda_{2}$ is close to the fixed amplitude ${\bar{h}}_{1}$, the noise reduction effect of double-wavelength serration is excellent, which is more conducive to the serrated geometry exacerbating the mutual destructive interference between nonlinear sound sources. When ${\bar{h}}_{1}=0.5$, there is no significant difference in the advantage of each serration relative to the double-wavelength sawtooth, and the SPL_CA_ values are almost close to zero, indicating that under this operating condition, the noise reduction ability of the double-wavelength sawtooth is no greater than single-wavelength serrations. As $\bar{h}$ increases, the advantages of double-wavelength serrations gradually emerge. It is worth noting that when $\bar{h}=2$, the sinusoidal double-wavelength serrations have the advantage of an additional SPL_CA_ = 2.4 dB over other types of serrated structures. Additionally, the noise reduction superiority of double-wavelength serrations is extremely sensitive to the wavelength $\lambda_{2}$. In brief, there is enough additional noise reduction potential with the appropriate double wavelength setting.

## 6. Results and Discussion

### 6.1. The Behavior of the Functions ${\left|E_{n}\right|}^{2}$

**Figure 27:**
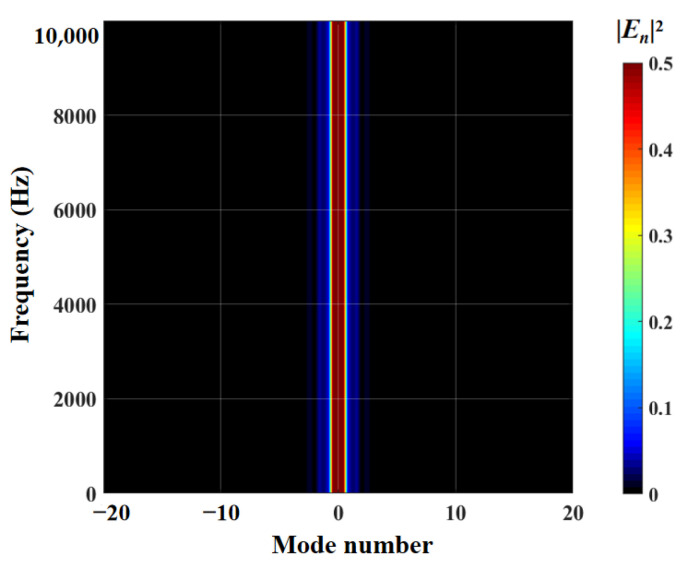
Cloud chart of ${\left|E_{n}\right|}^{2}$ built for the smooth leading edge.

**Figure 28:**
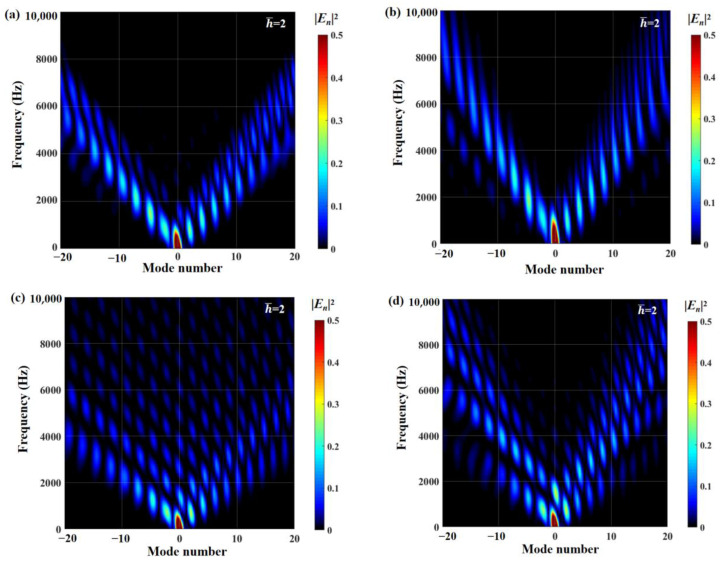
Cloud charts of ${\left|E_{n}\right|}^{2}$ for different double-wavelength serrations at $\bar{h}=$2, including (**a**) traditional serrations, (**b**) ogee-shaped serrations, (**c**) sinusoidal serrations, and (**d**) iron-shaped serrations.

The radiation integral ${\left|E_{n}\right|}^{2}$ related to the frequency and modal order play a significant role in qualitatively judging the acoustic energy radiation of a serrated structure. The distributions of the radiation integral ${\left|E_{n}\right|}^{2}$ related to the smooth leading edge and double-wavelength serrations are exhibited in Figure 27 and Figure 28. For the smooth leading edge, it is clear that only the mode N=0 takes effect in acoustic propagation. For double-wavelength serrations, the modal order gradually increases as the frequency increases. The modal amplitude decreases and the modal distribution is wider. In addition, all investigated serrations are dominated by the distribution of radiative integrals ${\left|E_{n}\right|}^{2}$ in modes of −10 to 10 orders.

Regarding sinusoidal serrations, modal scattering, whether in the low-frequency or high-frequency stages, possesses a small radiation integral. Simultaneously, compared to other serrations, the frequency-dependent modal scattering of sinusoidal serrations decays more rapidly at $\bar{h}=2$. In combination with the OASPL, the sinusoidal function is the best shape for overall noise reduction among the several types of serrations studied. Howe [30] mentions that ∂F(η)/∂η>1 is necessary for effective serrations. The region of ogee-shaped serrations with lower derivative ∂F(η)/∂η is smaller than other serrated airfoils, so it performs worst in the overall radiation integral, with the smallest degree of modal scattering. Due to the strong penetration of the lower-frequency region noise, scattering attenuation slowing down is more harmful to the human body, and secondly, the sound pressure level of the middle and low frequencies is the key to affecting the total sound pressure level. Therefore, more attention is paid to the distribution of radiation integrals in the dominant mode.

### 6.2. Sound Pressure Level (SPL)

**Figure 29:**
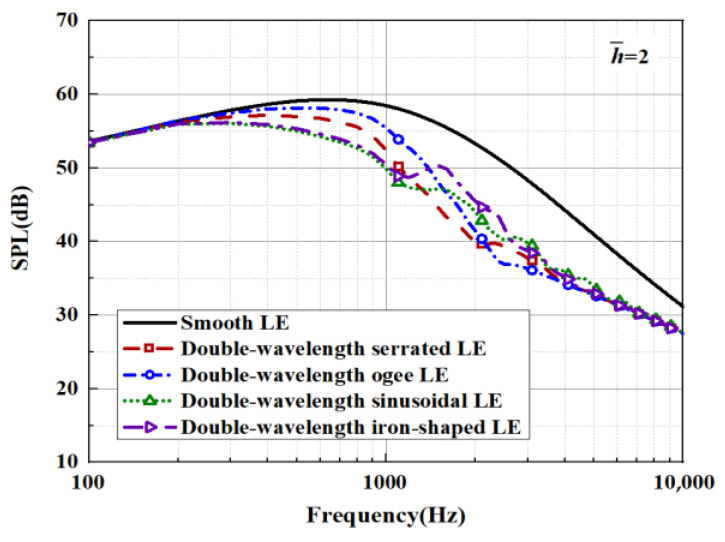
SPL spectra of representative double-wavelength serrations at $\bar{h}=$ 2.

**Figure 30:**
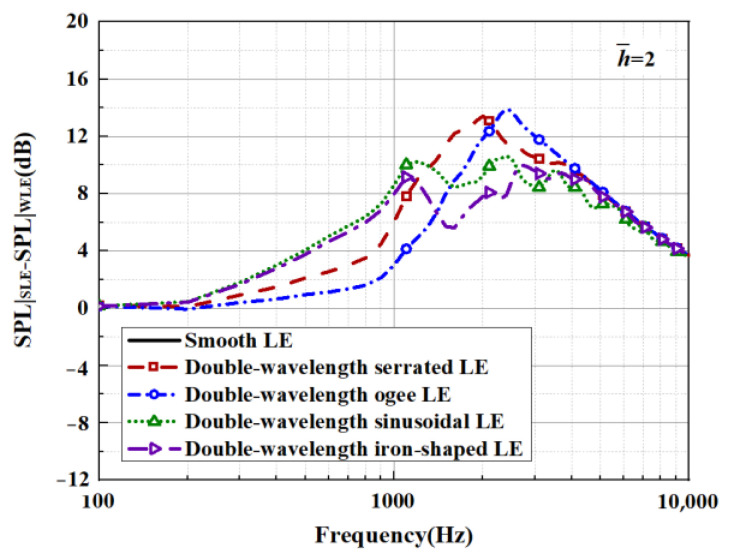
Sound pressure reduction levels of representative double-wavelength serrations at $\bar{h}=$ 2.

When evaluating the sound pressure spectrum for a given $k_{1}$, $\left|k_{3}\right|<\left|k_{1}\right|$ must be ensured. On the one hand, this is in order to match the turbulence generated in the experiment, and on the other hand, it is to ensure a finite acoustic wave assumption in the spanwise direction regarding a streamwise semi-infinite flat plate. The analytical far-field acoustic spectra are depicted in the case of $M=0.17,\theta =90^{{}^{\circ}}$ and *r* = 10. The analytical SPL spectrum of the smooth leading-edge serrations is solved by c = 0.001. Additionally, the analytical noise reduction levels of the representative double-wavelength serrations generated by the above trials are plotted in Figure 29 and Figure 30 under the condition of $\bar{h}=$ 2, respectively. Serrations play an important role in suppressing low- and intermediate-frequency noise. A noise reduction level of 14 dB at the specific frequency can be achieved. The noise reduction level shows a tendency to increase first and decrease later over the entire frequency band, showing good agreement with the laws of data obtained by Lyu [22].

### 6.3. Sound Pressure Level Integrated with Different Frequency Bands

**Figure 31:**
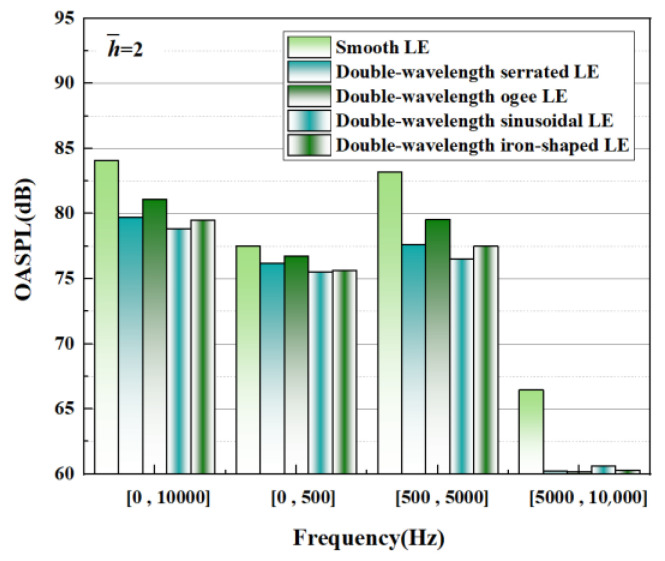
Integration of OASPLs of representative double-wavelength serrations over different frequency bands at $\bar{h}=2$.

According to the classification principle of the low-frequency, intermediate-frequency, and high-frequency bands, the frequency bands were divided into three representative frequency bands. The OASPLs integrated with different frequency bands are described in Figure 31. On the whole, a positive contribution induced by the leading-edge serrations is conducive to decreasing the OASPL to varying degrees, manifesting in the intermediate frequency band. Significantly, the intermediate-frequency bands are dominant in the OASPL, which corresponds to the frequency band with the largest broadband noise. This means that the reduction in the OASPL is closely related to the reduction in maximum broadband noise. In particular, the overall sound pressure reduction level is up to 5.19 dB in $\bar{h}=2,$ and the sound pressure level in the intermediate-frequency band is decreased by 6.71 dB. Except for this, the noise reduction ability of the different serrations is consistent in the order of noise reduction ability at the same $\bar{h}$. This illustrates the similarity of the noise reduction mechanisms of the serrated structures at different $\bar{h}$. The detailed noise reduction mechanisms are explored in the subsequent section.

### 6.4. Surface Pressure and Phase Distributions

The surface pressure is calculated as

(24) $$p_{s}\left(x,y,0_{+}\right)=\sum_{n=-\infty }^{\infty }\frac{1}{2\pi \beta \sqrt{-{\bar{k}}_{1}-w_{n}}}\int_{-\infty }^{\infty }\frac{e^{-\frac{i\lambda x}{\beta }}E_{n}\left(\lambda \right)}{\sqrt{\lambda +w_{n}}}d\lambda e^{ik_{2}y+2n\pi iy}e^{-\frac{ik_{1}M^{2}x}{\beta^{2}}}.$$

Single-frequency gusts were selected to simplify the research objective, which manifested in the replacement of a fixed wave number *k*_2_ with its integral over the wave number spectrum. This is conducive to clarifying the oblique-gust effect on surface pressure and interference and redistribution mechanisms. Surface pressures that act closely with far-field noise were calculated at $x=\bar{h}F(z)$. This corresponds to the geometry profile of the leading-edge serrations. Furthermore, a finite number of modes are summed, restricting the degree of difficulty of infinite modes to solve.

**Figure 32:**
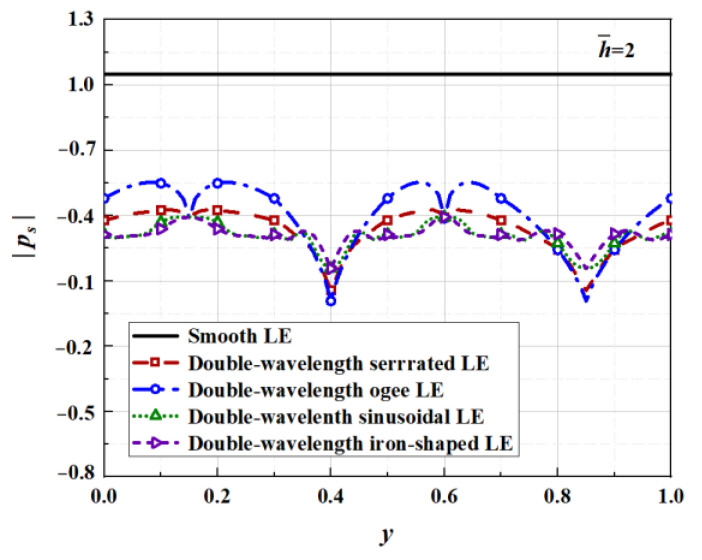
Surface pressure generating outgoing acoustic waves for double-wavelength serrations at $\bar{h}=$ 2.

Kim et al. [12] proposed a noise reduction mechanism for wavy leading edges adopting numerical simulation methods combined with various statistical analysis methods. The components of self-noise were eliminated by solving the full three-dimensional Euler inviscid solution. Unlike a straight leading edge, geometric obliqueness caused by the wavy leading edge led to the source cut-off effect of surface pressure fluctuations. In addition, the phase interference effect generated between the peak and hill center of the serrations contributed to noise reduction in the low- to mid-frequency range. The surface pressure and phase distribution were employed to further elucidate the two noise reduction mechanisms in this paper. According to the setting of the coordinate system of the studied flat plate, the parts of the negative value for F(η) in Figure 1 correspond to the peak of the serrations. On the contrary, the parts of the positive value denote the trough of the serrations. Combined with the following pressure distribution exhibited in Figure 32, it can be concluded that the surface pressure at the trough is greater than that at the peak.

The surface pressures along the wavy leading edge subjected to impinging turbulence were solved for $k_{3}=0$ at a specific frequency of $k_{1}=62.83$. In general, the absolute values of the surface pressure decrease gradually with the increase in $\bar{h}$. The maximum differential pressure from 1 to 0.55 achieves different degrees of acoustic energy attenuation. This verified that the source cut-off effect had taken effect. Additionally, the distributions of the absolute surface pressure are closely relevant to the serration profiles. The acoustic energy is reconstructed from the low propagating modes to higher cut-off modes. Source correlation is minimized at the serrated roots, resulting in a more significant reduction in the pressure distribution at the roots than in other locations.

**Figure 33:**
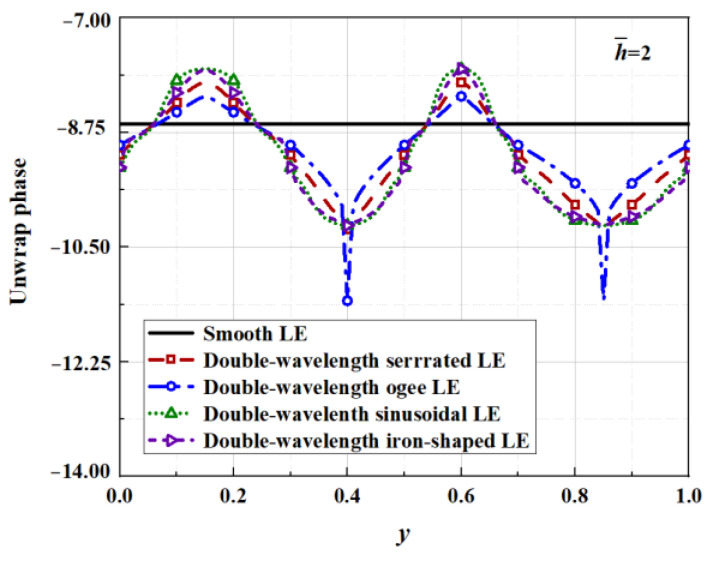
Phase distribution of surface pressure along the spanwise-varying leading edge of double-wavelength serrations at $\bar{h}=$ 2, M = 0.17, k_1_ = 62.83, k_3_ = 0.

Similarly, the noise reduction mechanism connected with phase interference is illustrated in Figure 33. The phase change at different amplitudes is similar to the geometrical profile. Moreover, the phase interference difference at the root of the ogee-shaped serration is in stark contrast to the other three serrations for $\bar{h}=2$, where spike-like depressions emerge. Therefore, the local phase difference is reduced by an additional maximum of 1.15 for $\bar{h}=2$. Nevertheless, the overall phase interference difference is not significantly weakened. Combined with the surface pressure along the leading edge, the phase difference between the traditional sawtooth and the sinusoidal serrations at the root is also smaller than the sinusoidal sawtooth and the iron-shaped serrations, indicating that a larger local phase difference can restrain the surface pressure of the outgoing sound energy.

It is speculative to reach a conclusion regarding the noise-reducing serration profiles. Compared with traditional serrations and ogee-shaped serrations, the sinusoidal serrations and iron-shaped serrations form a larger inclination angle with the *z*-axis, and both of them have convexity-preserving properties. Moreover, the ogee-shaped serrations contribute to the local phase interference difference. It can be inferred that serrations combined with the above two advantages have more advantages in reducing the aeroacoustic noise emitted by surface pressure. The trade-off between the pressure field formed by the destructive interference and the variable pressure field between the root and the hill can result in a more excellent noise abatement effect.

### 6.5. Directivity Characteristic of OASPL

**Figure 34:**
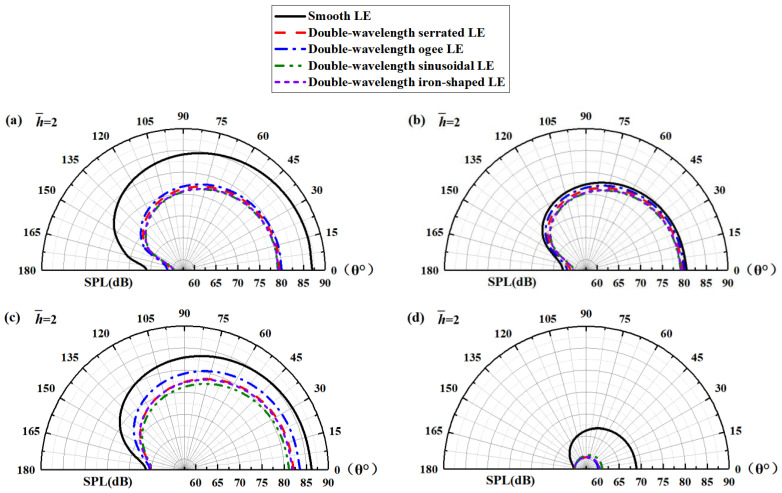
Spatial distribution of OASPL integrated over the frequency bands of (**a**) 0~10,000 Hz, (**b**) 0~500 Hz, (**c**) 500~5000 Hz, and (**d**) 5000~10,000 Hz at $\bar{h}=$ 2.

Judging from the above analysis, it can be deduced that noise at a specific frequency does not reflect the change law of the overall sound pressure level very well unless enough frequency bands are taken. So, the broadband noise integrated over different frequency bands is likely responsible for the aerodynamic noise reduction. Combined with the above frequency band division basis, the broadband noise in each band was analyzed. To describe the relationship between the additional noise reduction gain of the double-wavelength serrations and the observer angles, the SPL directionality mode of the smooth airfoil and the double-wavelength serrations was compared, as depicted in Figure 34. The directivity of the sound pressure level was not changed by the leading-edge serrations. It is worth noting that additional sound suppression of double-wavelength serrations is achieved at almost all observer angles. For the sinusoidal serration profile of the optimal noise reduction level at low and medium frequencies, there was some increase in the undesirable noise in high frequency, but the negative contribution to the reduction in the overall sound pressure level was negligible. As a consequence, noise reduction for broadband noise in low-to-intermediate frequencies is critical to diminish the overall sound pressure level.

## 7. Conclusions

The noise reduction performance of a double-wavelength serration was analyzed in this study, and a systematic parameterization study was carried out to determine the matching degree of the optimal wavelength and amplitude for the maximum noise reduction level. The conclusions obtained are summarized as follows.

(1) Combined with the function $\left|E_{n}\right|$ associated with the shape factor, noise reduction at specific frequencies can be obtained in the low- and intermediate-frequency bands for serrations with a small curvature at non-smooth points. Moreover, it should be mentioned that spanwise-varying leading edges coping with a subsonic airflow interact with sharp serrations, resulting in an increase in additional broadband noise in the low-frequency regime compared with traditional serrations. And the performance in the high-frequency region is reversed. However, when the influence of trailing-edge self-noise is gradually enhanced with the increase in $\bar{h}$, the noise reduction advantage of large-curvature serrations at non-smooth points in the high-frequency region is weakened to a certain degree. For a single-value piecewise function in the unit wavelength range of 1 and 1/4 period, one must meet the $\left|\partial F(\eta )/\partial \eta \right|>4\bar{h}$ condition in order to design a serration with excellent noise reduction performance at low and intermediate frequencies, while the opposite represents a good noise reduction level in the high-frequency region.
(2) Aside from this, the acoustic optimality of double-wavelength serrations of different frequencies and phases is the key focused discussion. The noise reduction levels of shape factors, wavelengths, and amplitudes at different tip-to-root ratios are obtained through the design of 16×4 trial numbers. It turns out that the shape factor was dominant at different tip-to-root ratios. The amplitude gradually replaces the wavelength with the increase in the tip-to-root ratio and transforms into the second most influential factor. Based on the premise of fixed wavelength and amplitude, the noise reduction level at $x_{2}=1$ performed best. This indicates that the larger the amplitude of the superimposed serrations, the more conducive it is to increasing the phase difference.
(3) Both the sinusoidal function and the iron-shaped function performed well at different double-wavelength serrations, reducing the overall sound pressure level by up to 5.2 dB. Broadband noise in the 500–5000 Hz band was significantly reduced by 6.7 dB. The root-destructive interference was enhanced by the ogee-shaped serrations, increasing the local noise reduction effect and suppressing noise emissions in specific high-frequency regions. In the design of serrations, one can refer to the source cut-off mechanism of source radiation and introduce sharp structures such as narrow slits at the root to achieve local noise control, satisfying the design requirement of reducing high-frequency noise.

## Figures and Tables

**Table 1:** Trial design of different levels and factors.

Parameters	Shape	$\lambda_{2}$	$h_{2}$	$\lambda_{1}$	$h_{1}$
Factors	A	B	C	D	E
Level 1	Serrated	0.2	0.2	0.8	1
Level 2	Ogee	0.4	0.5	0.6	1
Level 3	Sinusoidal	0.6	0.8	0.4	1
Level 4	Iron-shaped	0.8	1	0.2	1

**Table 2:** The calculation results based on the trial design regarding $\bar{h}=1$.

Trial Number	Code Name	$\lambda_{2}$	$h_{2}$	SPL (dB)	SPL_1_	SPL_2_	SPL_CA_	Results
1	A_1_B_1_C_1_	0.2	0.2	81.3600	−0.3541	−2.3381	−1.3461	I1
2	A_1_B_1_C_2_	0.2	0.5	81.2934	−0.4207	−1.2235	0.8221	I_2_
3	A_1_B_1_C_3_	0.2	0.8	81.2534	−0.4607	0.0759	0.1924	I_3_
4	A_1_B_1_C_4_	0.2	1	81.2595	−0.4546	0.9309	0.2382	I_4_
5	A_1_B_2_C_1_	0.4	0.2	81.5507	0.7586	−2.2048	0.7231	I_5_
6	A_1_B_2_C_2_	0.4	0.5	81.2268	0.4347	−1.4466	0.5060	I_6_
7	A_1_B_2_C_3_	0.4	0.8	80.7423	−0.0498	−0.5733	−0.3116	I_7_
8	A_1_B_2_C_4_	0.4	1	80.4740	−0.3181	−0.0542	−0.1862	I_8_
9	A_1_B_3_C_1_	0.6	0.2	82.3493	1 8211	−1.4556	0.1828	I_9_
10	A_1_B_3_C_2_	0.6	0.5	81.9631	1.4349	−0.9077	0.2636	I_10_
11	A_1_B_3_C_3_	0.6	0.8	81.0968	0.5686	−0.4463	0.0612	I_11_
12	A_1_B_3_C_4_	0.6	1	80.4483	−0.0799	−0.3438	−0.2119	I_12_
13	A_1_B_4_C_1_	0.8	0.2	83.1816	2.8530	−0.7657	1.0437	I_13_
14	A_1_B_4_C_2_	0.8	0.5	82.8609	2 5323	−0.2522	1.1401	I_14_
15	A_1_B_4_C_3_	0.8	0.8	81.9630	1.6344	−0.1386	0.7479	I_15_
16	A_1_B_4_C_4_	0.8	1	81.1964	0.8678	−0.5177	0.1751	I_16_
17	original	—	—	84.0530	—	—	—	—

**Table 3:** The multivariate analysis of the different types of serrations regarding $\bar{h}=0.5,\;1$ and 2.

Factors	A	B	C
$R_{\bar{h}=05}$	0.809	0.342	0.171
$R_{\bar{h}=1}$	3.092	1.096	1.027
$R_{\bar{h}=2}$	4.301	1.481	1.627
Order of the factors ($\bar{h}=0.5$)	A > B > C		
Order of the factors ($\bar{h}=$1)	A > B > C		
Order of the factors ($\bar{h}=$2)	A > C > B		

## Data Availability

Data will be made available on request.
